# Supplementary material for: Obesity-Altered Adipose Stem Cells Promote ER+ Breast Cancer Metastasis through Estrogen Independent Pathways
Source: Int J Mol Sci. 2019 Mar 20;20(6):1419. doi: 10.3390/ijms20061419 (PMC6470828; doi:10.3390/ijms20061419)
Supplement: Supplementary file 1 [file ijms-20-01419-s001.zip › ijms-459231-supplementary-final.pptx]

## Slide 1
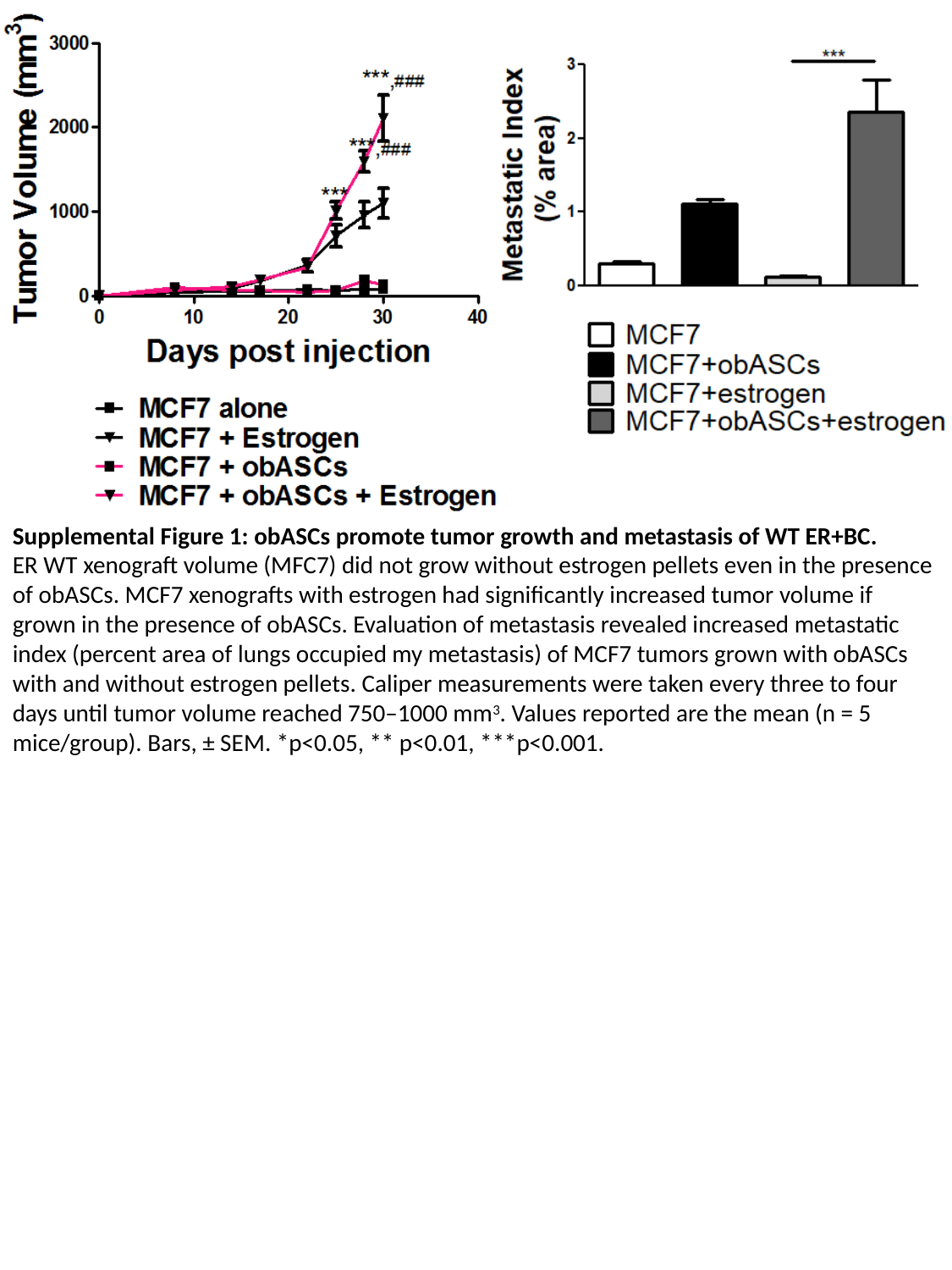

Supplemental Figure 1: obASCs promote tumor growth and metastasis of WT ER+BC.
ER WT xenograft volume (MFC7) did not grow without estrogen pellets even in the presence of obASCs. MCF7 xenografts with estrogen had significantly increased tumor volume if grown in the presence of obASCs. Evaluation of metastasis revealed increased metastatic index (percent area of lungs occupied my metastasis) of MCF7 tumors grown with obASCs with and without estrogen pellets. Caliper measurements were taken every three to four days until tumor volume reached 750–1000 mm3. Values reported are the mean (n = 5 mice/group). Bars, ± SEM. *p<0.05, ** p<0.01, ***p<0.001.

## Slide 2
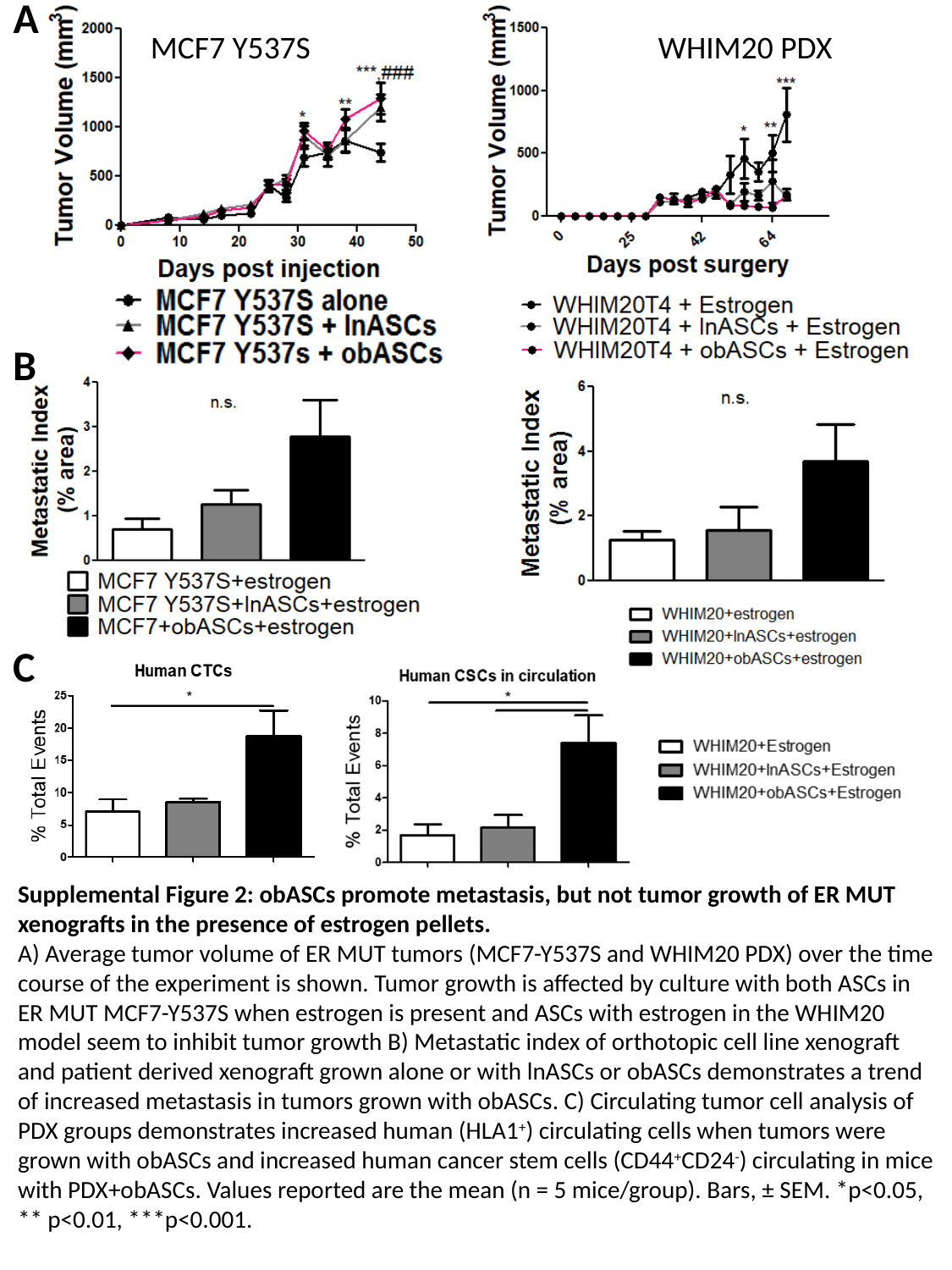

A
MCF7 Y537S 			WHIM20 PDX
B
C
Supplemental Figure 2: obASCs promote metastasis, but not tumor growth of ER MUT xenografts in the presence of estrogen pellets.
A) Average tumor volume of ER MUT tumors (MCF7-Y537S and WHIM20 PDX) over the time course of the experiment is shown. Tumor growth is affected by culture with both ASCs in ER MUT MCF7-Y537S when estrogen is present and ASCs with estrogen in the WHIM20 model seem to inhibit tumor growth B) Metastatic index of orthotopic cell line xenograft and patient derived xenograft grown alone or with lnASCs or obASCs demonstrates a trend of increased metastasis in tumors grown with obASCs. C) Circulating tumor cell analysis of PDX groups demonstrates increased human (HLA1+) circulating cells when tumors were grown with obASCs and increased human cancer stem cells (CD44+CD24-) circulating in mice with PDX+obASCs. Values reported are the mean (n = 5 mice/group). Bars, ± SEM. *p<0.05, ** p<0.01, ***p<0.001.
